# The effectiveness of walking exercise on the bowel preparation before colonoscopy: a single blind randomized clinical trial study

**DOI:** 10.1186/s12876-023-02987-x

**Published:** 2023-10-09

**Authors:** Gholamreza Rezamand, Farahnaz Joukar, Ehsan Amini-Salehi, Hamed Delam, Reza Zare, Alireza Samadi, Sara Mavadati, Soheil Hassanipour, Fariborz Mansour-Ghanaei

**Affiliations:** 1 Gastrointestinal and Liver Diseases Research Center, Guilan University of Medical Sciences, Rasht, Iran; 2 Student Research Committee, Larestan University of Medical Sciences, Larestan, Iran; 3 University Hospital of Bari, Bari, Italy

**Keywords:** Walking exercise, Colonoscopy, Bowel preparation, Randomized control trial, Gastrointestinal cancer, Bowel enhancement

## Abstract

**Background and Aim:**

Bowel preparation is a crucial factor affecting the diagnostic accuracy of colonoscopy, and few randomized control trials evaluated enhancement in bowel preparation. In this study, we aimed to evaluate the effectiveness of walking exercises on bowel preparation before a colonoscopy procedure.

**Methods:**

The present study is a single-blind randomized controlled trial involving 262 patients scheduled for colonoscopy procedures. These patients were randomly assigned to two groups: an intervention group (n = 131) and a control group (n = 131). In the intervention group, participants followed a predetermined plan that included the consumption of specific liquids and foods, bisacodyl pills, polyethylene glycol powder, and a regimen of walking exercises in preparation for their colonoscopy. Conversely, individuals in the control group followed the same regimen but were not instructed to engage in walking exercises. On the day of the colonoscopy, both groups were assessed for their level of physical activity using a foot counter. Additionally, an experienced gastroenterologist evaluated and compared the bowel preparation between the two groups using the Boston Bowel Preparation Scale (BBPS).

**Results:**

The number of footsteps recorded in the two groups exhibited a significant difference (P < 0.001). Although there was no statistically significant difference between the intervention and control groups in terms of mean BBPS scores (6.26 ± 1.9 vs. 6.29 ± 1.9, P = 0.416), individuals who took more than 6900 steps had significantly higher BBPS scores compared to those with fewer than 6900 footsteps (6.62 ± 1.8 vs. 5.92 ± 1.9, P = 0.003).In the univariate analysis, BBPS was found to be significantly associated with individuals under the age of 50 (OR: 2.45, 95% CI: 1.30–4.61, P = 0.006) and smoking status (OR: 0.41, 95% CI: 0.17–0.94, P = 0.043). In the multivariate analysis, the relationship between BBPS and age below 50 and smoking remained significant (OR: 2.50, 95% CI: 1.30–4.70, P = 0.005, and OR: 0.38, 95% CI: 0.16–0.93, P = 0.034, respectively).

**Conclusion:**

A higher number of footsteps taken especially more than 6900 can significantly enhance bowel preparation; however, walking exercise as an intervention before colonoscopy is not significantly associated with BBPS. Also, older people and smokers seem to have fewer benefits from walking exercises for bowel preparation.

**Trial registration:**

ISRCTN32724024 (Registration date:22/08/2018).

## Background

Colorectal cancers are widely recognized as among the most harmful forms of cancer worldwide [1–4]. International reports reveal that colorectal cancer ranks as the third most prevalent cancer and is also the third leading cause of cancer-related mortality across both genders [5]. Several risk factors have been associated with colorectal cancers, including low levels of physical activity, a high-fat diet, a high body mass index (BMI), limited consumption of vegetables and fruits, smoking, alcohol, consumption, and a family history of the disease [4, 6].

Screening for this cancer and early detection and removal of neoplastic colon polyps can reduce colorectal cancer mortality [7, 8]. Studies have shown that colonoscopy is the most effective screening and diagnostic method in colorectal cancer management, and its usage has been expanding dramatically in recent years [7, 9–12]. Colonoscopy provides the detection and removal of suspicious lesions, enhancement of adenoma detection rate, and decrease in colon cancer risk [13].

Intestinal cleansing is one of the crucial determinants of the operators’ performance during colonoscopy [14]. Better bowel preparation results in higher intestinal cleanliness providing the operators with a better view of possible lesions in the lumen [15]. In contrast, poor bowel preparation results in missing the detection of the lesions and a longer duration of the procedure [16, 17].

The current bowel preparation guidelines before colonoscopy recommend administering intestinal cleansers like polyethylene glycol (PEG), sodium phosphate, and simethicone 4–6 h before the procedures [18, 19].

Previous animal studies have shown that exercising can ease gastrointestinal (GI) system motility and better defecation [20, 21]; hence it can be hypothesized that mild walking exercise for outpatients can enhance their colonoscopy efficiency by increasing bowel cleanliness. However, limited clinical trial studies with controversial results have been performed on the effects of planned walking exercise on colon cleansing in colonoscopy [22, 23]; therefore, we performed this single-blind randomized study to identify exercise’s effect on bowel preparation before colonoscopy.

## Materials and methods

### Study design and setting

The present study is a single-blind randomized clinical trial (RCT) with an allocation ratio of 1:1. The study protocol was registered in International Standard Randomized Controlled Trial Number (ISRCTN) (Registration number: ISRCTN32724024, Registration date: 22/8/2018). The study population included the patients who were referred to Razi Hospital (Rasht, Guilan province, Iran) by a Gastroenterologist for colonoscopy procedures for diagnostic indications from June 2018 up to October 2018.

Patients with the following criteria were eligible for the study: (1) age was between 18 and 70 years old regardless of gender; (2) being aware of the study protocols or having an alert companion. Patients were excluded from the study if they had the following criteria: (1) showing allergic reactions to the drugs used in the study; (2) being a pregnant or lactating woman; (3) having underlying diseases that make the exercise uncomfortable for the patient, like heart, lung, malignant diseases, and diabetes mellitus; (4) having hip and knee joint replacement or any movement problems.

We used random sampling (Random Allocation) using the online software to allocate participants into two intervention and control groups. All patients were randomly divided into two groups of intervention (having walking exercise) and control (without walking exercise).

### Bowel preparation protocol

The bowel preparation protocol was explained to the patients by a trained researcher. To ensure the correctness of the intervention, two phone calls were made to all participants on the day before the colonoscopy procedure. Written consent was obtained from patients to participate in the project. At each stage of the study, patients could leave the study at their discretion.

The protocol of bowel preparation before colonoscopy was as follows:

### The day before the colonoscopy

The intervention group participants were supposed to eat a light breakfast at 8 a.m., without milk and other dairy products. From 8 a.m. to 12 p.m., they were allowed to drink only clear liquids in the amount of 8 to 10 glasses (such as water, tea, or lemon juice) and walk for 5 min. From 12 p.m. to 1 p.m., they were told to eat a light lunch (like a piece of cooked chicken with a slice of bread) and two bisacodyl pills and then walk for 5 min. Then the patients were not allowed to eat any solid food until the colonoscopy procedure. The patients were told to dissolve three packs of PEG in 3 L of water (approximately equivalent to 12 to 13 full glasses) drink about one glass of this solution (the first PEG solution) every 15 to 20 min from 3 p.m. and walk for 5 min.

The participants were supposed to eat two other bisacodyl pills at 6 p.m. and walk for 5 min. After finishing the first PEG solution, our patients were allowed to drink clear liquids such as water, tea, and completely strained fruit juice as much as they wanted and walk for 5 min. After finishing the first PEG solution, they were allowed to rest for about 2 h. They were supposed to dissolve two other packets of PEG powder in two liters of water (about eight glasses) and then drink them (the second PEG solution) from 9 p.m. every 15 to 20 min and walk for 5 min. After finishing the second PEG solution, the patients were forbidden to eat or drink anything until the colonoscopy.

The day before the colonoscopy for the control group was similar to the intervention group, but the participants were not told to walk.

Patients were given smart bands (Mi band 2, Xiaomi) and monitored to assess the number of footsteps taken. An alert patient companion observed all steps of the protocol for each patient.

### The colonoscopy procedure

On the colonoscopy day, all patients had the procedure by an experienced gastroenterologist and two trained nurses. The gastroenterologist and the nurses were blind regarding the group of patients. Propofin and etomidate were used as narcotic drugs. A Fuji 600 series scope with a processor model 4050 was utilized. No distal attachments were used during the procedures. Our approach involved the application of Fice chromoendoscopy and the utilization of water/air insufflation. Two trained assistants recorded the total procedure and the intubation time.

### Bowel preparation evaluation

Boston Bowel Preparation Scale (BBPS) was used to score bowel preparation [24]. In this criterion, the colon is divided into three parts, including 1-ascending colon, 2-transverse colon, 3-descending colon, and rectum [25]. Every part is scored from 0 to 3 based on the following:

Score 0: The colon was not visible without mucosal preparation due to the presence of solid stools.

Score 1: Some Parts of the mucous were seen in the colon, but others were not visible due to stains, stools, or opaque fluid.

Score 2: A small amount of residue, small pieces of stool, or opaque fluid existed, but the mucosal part of the colon was seen well.

Score 3: All mucosal layer of the colon was seen well without any stains, small pieces of stool, or opaque fluid.

The predictive scale used for bowel preparation was appropriate if BBPS score ≥ 2 was observed in each three parts of the colon.

### Data collection

A trained assistant at the gastroenterology and liver research center initially registered individuals. Then the socioeconomic characteristics, social history (drug use, smoking, and alcohol consumption), chief complaint (reason for referral), defecation status, history of gastrointestinal or gynecological surgery, history of chronic diseases, history of previous colonoscopies and the number of footsteps (recorded by the smart band) were completed by a trained questioner. Also, during the colonoscopy, the rest of the questionnaire was completed by two trained questioners, including the time of the colonoscopy and the time of reaching the cecum. A gastroenterologist ultimately determined the final diagnosis, Boston score, and future colonoscopy status.

### Data analysis

Data analysis was done using SPSS version 21 statistical software. In order to investigate the difference between the two intervention and control groups, the χ2 test and student t-test were used for categorical variables. One-way ANOVA test was used to evaluate the difference between bowel cleanliness scores in the two groups. In addition, univariate or multivariate regression analysis was used to find the influencing factors in bowel cleansing. A significance level was considered as P < 0.05. The sample size in each group was calculated at 131 people according to the prior pilot study and the mean ± SD for the intervention and control group (6.5 ± 1.8 and 5.6 ± 3.06, respectively).

$$\frac{{{(Z}_{1-\frac{{\upalpha }}{2}}+{\text{Z}}_{1-{\upbeta }})}^{2}\left[{\text{S}}_{1}^{2}+{\text{S}}_{2}^{2}\right]}{{\left({{\upmu }}_{1}-{{\upmu }}_{2}\right)}^{2}}\cong 131$$

$$\alpha = 0.05\,effective\,size\,:\,0.5$$

$$\beta = 0.1$$

## Results

### Base line characteristics

**Fig. 1:**
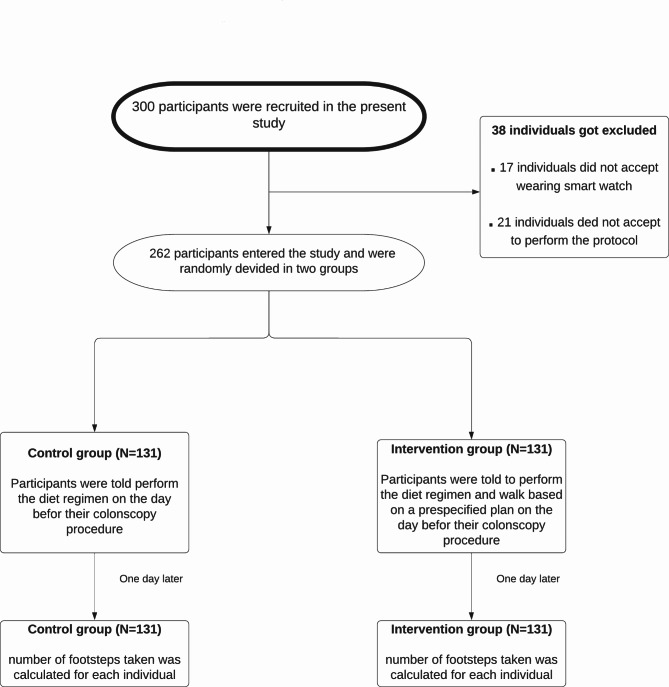
Consort flowchart of study

A total of 300 participants were recruited to contribute to the study. Of these, 38 individuals were excluded for various reasons, like failing to perform the trial protocol or not using the smart band. A total of 262 patients were finally divided into two groups of intervention (n = 131) and control (n = 131) (Fig. 1). The mean ages of the intervention and control groups were 50.90 ± 14.27 and 51.70 ± 14.10 years old, respectively. The basic information of the participants in the intervention and control groups is shown in Table 1. No significant difference was observed between the intervention and control groups except for the number of footsteps taken (8866.20 ± 4699.00 vs. 6120.60 ± 3975.00, *P* < 0.001). The trial was ended on 19 October 2018.

**Table 1:** Baseline information of the intervention and the control groups

Variable	Intervention group (n = 131)	Control group (n = 131)	*P*-value
Age, mean ± SD^*^	50.90 ± 14.27	51.70 ± 14.10	0.621
Gender, % of men	58 (9/42)	67 (2/49)	0.298
Number of steps, mean ± SD	8866.20 ± 4699.00	6120.60 ± 3975.00	< 0.001
BMI^**^, mean ± SD	26.70 ± 4.50	26.80 ± 5.90	0.878
Smoking, % of positive responses	11 (8.10)	16 (11.70)	0.320
History of gastrointestinal surgery, % of positive responses	22 (16.20)	20 (14.70)	0.718
History of gynecological surgery, % of positive responses	34 (25.10)	36 (26.40)	0.809
History of chronic disease, % of positive responses	59 (43.70)	69 (50.70)	0.223
Colonoscopy time (min)	20.20 (11.00)	18.30 (9.00)	0.123
Time to receive cecum (min)	9.80 (6.50)	9.00 (6.80)	0.329

^*^Standard Deviation, ^**^Body Mass Index

### Primary outcomes

#### Bowel cleanliness

Our results showed that although there was no significant difference between the intervention and control groups regarding mean BBPS (6.26 ± 1.9 vs. 6.29 ± 1.9, *P* = 0.885), there was a significant difference between the individuals with less than 6900 steps and more than 6900 steps. The mean BBPS score in the individuals with more than 6900 steps was significantly higher than individuals with less than 6900 steps (6.62 ± 1.8 vs. 5.92 ± 1.9, *P* = 0.003) (Table 2).

**Table 2:** BBPS in different groups

Groups	Mean ± SD	*P* value
Intervention group	6.26 ± 1.9	0.885
Control group	6.29 ± 1.9	
Individuals higher than 6900 footsteps	6.62 ± 1.8	0.003
Individuals less than 6900 footsteps	5.92 ± 1.9	

### Secondary outcomes

#### Results of univariate analysis

**Fig. 2:**
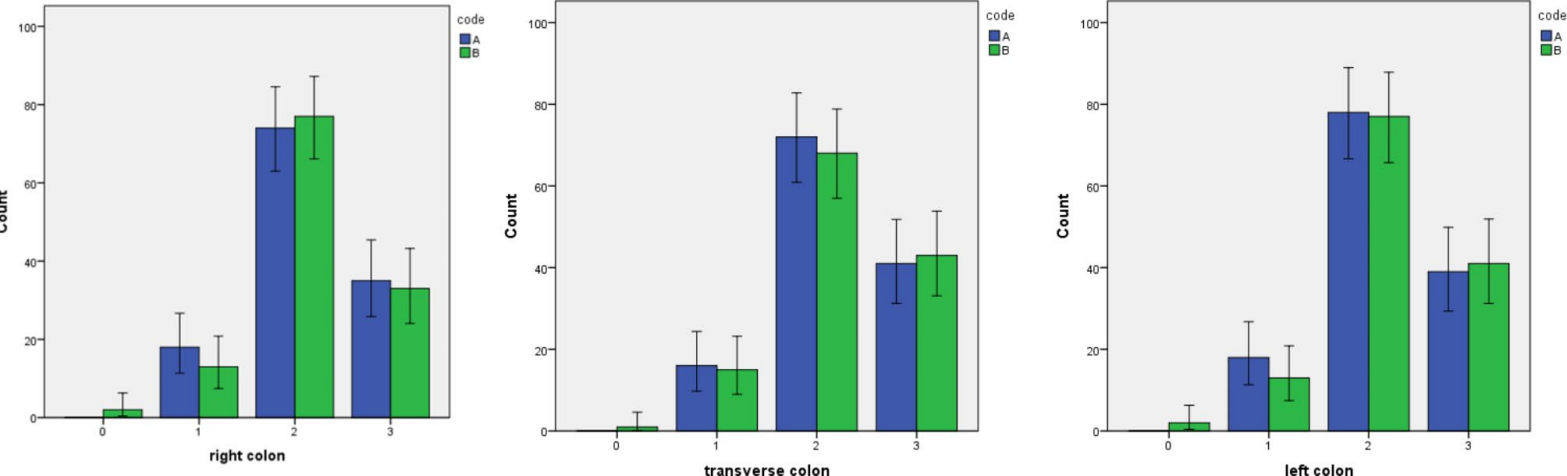
Boston Bowel Preparation Score (BBPS) in two groups of intervention (Blue bar) and control (green bar)

The results of univariate regression analysis related to the factors affecting bowel cleansing (BBPS higher than 5 vs. less than 5) showed no significant correlation between gender (*P* = 0.125), walking exercise (*P* = 0.416), BMI (*P* = 0.904), diarrhea (*P* = 0.201), constipation (*P* = 0.399), history of chronic diseases (*P* = 0.051), history of gastrointestinal (*P* = 0.377) or gynecological surgeries (*P* = 0.881) with high BBPS. However, the association between BBPS with age less than 50 (*P* = 0.006) and smoking was significant (*P* = 0.043) (Table 3; Fig. 2).

The results of univariate regression analysis related to the factors affecting right colon cleansing (BBPS higher than 2 vs. less than 2) showed no significant correlation between gender (*P* = 0.318), walking exercise (*P* = 0.609), BMI (*P* = 0.931), diarrhea (*P* = 0.127), constipation (*P* = 0.413), history of chronic diseases (*P* = 0.107), history of gastrointestinal (*P* = 0.903) or gynecological surgeries (*P* = 0.791) and smoking (*P* = 0.060) with high BBPS. However, the association between BBPS with age less than 50 (*P* = 0.004) was significant (Table 3; Fig. 2).

The results of univariate regression analysis related to the factors affecting left bowel cleansing (BBPS higher than 2 vs. less than 2) showed no significant correlation between age (*P* = 0.063), walking exercise (*P* = 0.609), BMI (*P* = 0.827), diarrhea (*P* = 0.298), constipation (*P* = 0.648), history of chronic diseases (*P* = 0.874), history of gastrointestinal (*P* = 0.980) or gynecological surgeries (*P* = 0.293) with high BBPS. However, the association between BBPS with smoking (*P* < 0.001), and male gender (*P* = 0.014) was significant (Table 3; Fig. 2).

**Table 3:** Univariate analysis logistic regression analysis predicting factors affecting total BBPS higher than 5, right colon BBPS higher than 2, left colon BBPS higher than 2

Variable	Univariate Analysis^*^ of total BBPS higher than 5			Univariate Analysis^*^ of BBPS higher than 2 in right colon			Univariate Analysis^*^ of BBPS higher than 2 in left colon		
	OR^‡^	95% CI	*P* value	OR^‡^	95% CI	*P* value	OR^‡^	95% CI	*P* value
Gender Man Woman	0.63	(0.35 − 0.113)	0.125	0.68	(0.33–1.43)	0.318	0.38	(0.17–0.82)	0.014
Age, year < 50 ≥ 50	2.45	(1.30–4.61)	0.006	3.92	(1.55–9.88)	0.004	2.15	(0.96–4.83)	0.063
Walking Yes No	1.27	(0.71–2.26)	0.416	1.21	(0.58–2.52)	0.609	1.21	(0.58–2.51)	0.609
BMI, kg/m^2^ < 25 ≥ 25	0.96	(0.53–1.73)	0.904	0.96	(0.45–2.04)	0.931	1.08	(0.51–2.27)	0.827
Diarrhea Yes No	1.82	(0.72–4.56)	0.201	3.15	(0.72–13.74)	0.127	1.92	(0.56–6.64)	0.298
Constipation Yes No	0.77	(0.43–1.39)	0.399	0.73	(0.34–1.54)	0.413	1.18	(0.57–2.45)	0.648
Smoking Yes No	0.41	(0.17–0.94)	0.043	0.38	(0.13–1.04)	0.060	0.16	(0.06–0.41)	< 0.001
History of gastrointestinal surgery Yes No	1.47	(0.62–3.53)	0.377	1.06	(0.38–2.94)	0.903	1.01	(0.36–2.79)	0.980
History of gynecological surgery Yes No	1.05	(0.54–2.03)	0.881	0.89	(0.39–2.04)	0.791	1.64	(0.65–4.18)	0.293
History of chronic disease Yes No	0.56	(0.31-1.00)	0.051	0.54	(0.15–1.14)	0.107	0.94	(0.45–1.95)	0.874

^*^Pvalue based on the χ2 test, ^**‡**^**Odds Ratio**

#### Results of multivariate analysis

The result of multivariate analysis showed a significant relationship between age less than 50 and total BBPS higher than 5, right colon BBPS higher than 2, and left colon BBPS higher than 2 (*P* = 0.005, *P* = 0.004 and *P* = 0.043 respectively). The relation between smoking and total BBPS and left colon BBPS was significant (*P* = 0.034 and *P* < 0.001, respectively); however, it showed no significant effect on right colon BBPS (***P*** = 0.055) (Table 4).

**Table 4:** Multivariate logistic regression analysis of predicting factors affecting total BBPS higher than 5, right colon BBPS higher than 2, left colon BBPS higher than 2

Variable	Univariate Analysis^*^ of total BBPS higher than 5			Univariate Analysis^*^ of BBPS higher than 2 in right colon			Univariate Analysis^*^ of BBPS higher than 2 in left colon		
	OR^‡^	95% CI	*P*	OR^‡^	95% CI	*P*	OR^‡^	95% CI	*P*
Age, year < 50 ≥ 50 (Reference group)	2.50	(1.30–4.70)	0.005	3.98	(1.57–10.10)	0.004	2.39	(1.02–5.56)	0.043
Smoking Yes No (Reference group)	0.38	(0.16–0.93)	0.034	0.35	(0.12–1.02)	0.055	0.15	(0.06–0.38)	< 0.001

^*^P value based on the χ2 test; ^**^P value based on logistic regression, ^‡^ Odds Ratio

## Discussion

Colonoscopy is a great tool for assessing large intestine and rectum abnormalities. It can be used for evaluating many GI diseases such as bleeding hemorrhoids [26, 27], Crohn’s disease [28], ulcerative colitis [29], chronic diarrhea [30, 31], rectum prolapse [32], colon polyps [33, 34], colon cancer [35] and occult blood in feces [36]. Using colonoscopy to screen colorectal cancer can lead to less incidence and mortality in the general population [37, 38]. Colonoscopy is the most common method used for colorectal cancer screening in the United States [39].

Bowel preparation before colonoscopy is essential and determines the imaging quality [40]. The terms “excellent”, “good”, “fair”, and “poor” are used to describe the quality of bowel preparation [41–44]. Poor bowel preparation can lead to missing the pathologic lesions in colonoscopy and wrong diagnoses [45–48].

There are some factors which can determine the quality of bowel preparation including age [49, 50], gender [50–52], underlying diseases such as diabetes [53, 54], cerebrovascular accidents [51], prior surgery [53] and socioeconomic status [49, 50, 55]. Although preparation is important, it is usually unpleasant for patients because of the bad taste of the used agents [41, 56, 57]. The two agents that are commonly administered for the preparation of colonoscopy are polyethylene glycol (PEG) and sodium phosphate (NaP) [58, 59]. PEG is not well tolerated in patients undergoing colonoscopy because of the high volume and bad taste, while the safety of NaP, especially in patients with renal failure, is challenging [60]. It has also been reported that 19.7% and 16.4% of patients undergoing colonoscopy suffer abdominal pain and vomiting during bowel preparation, respectively [61].

Several items can lead to better preparation, including following instructions precisely [49, 50] and the time of bowel preparation [62–65]. Recent studies suggested that walking exercises before colonoscopy might be effective in bowel preparation [66, 67].

**Fig. 3:**
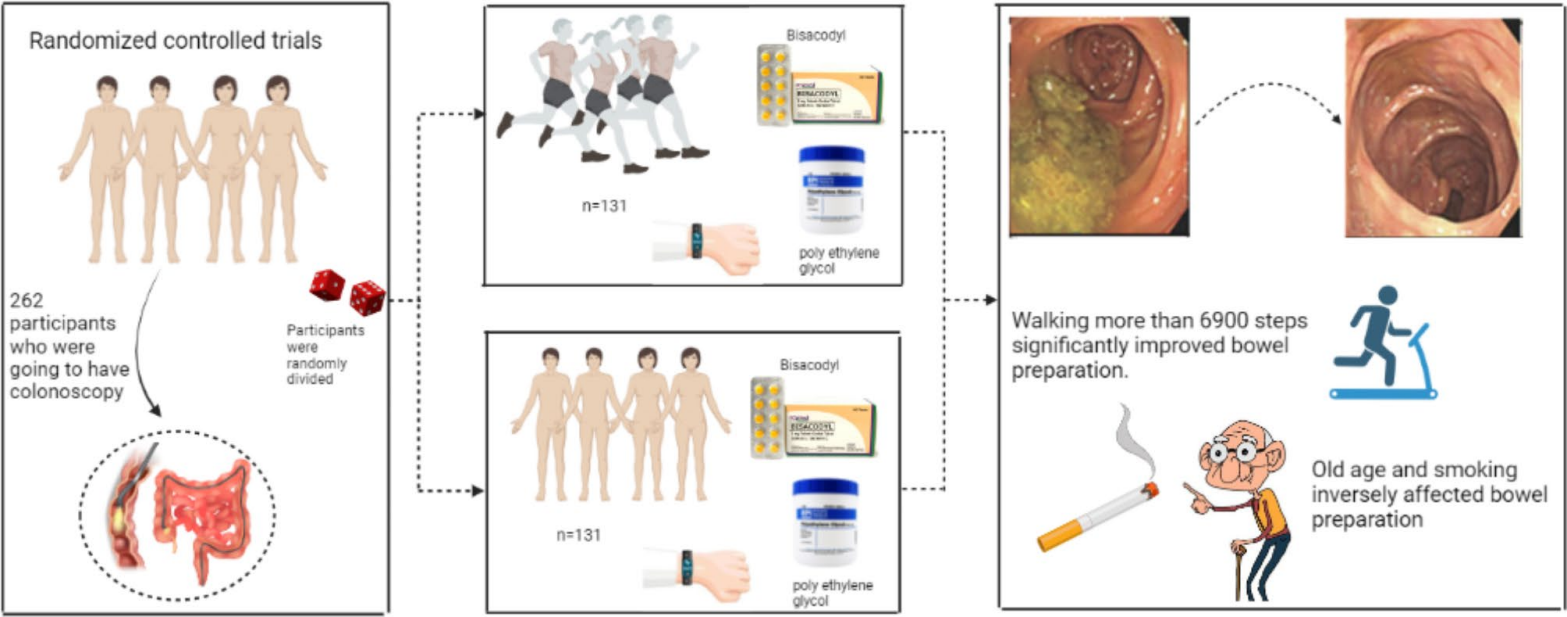
Summary of study setting and results

In this study, although there was not a significant correlation between walking and bowel cleansing in the intervention and control groups, individuals with more than 6900 steps had significantly higher bowel cleansing scores (Fig. 3). Another study by Zhang et al. showed that patients who walk longer (regarding walking time) have better bowel preparation [68]. Another study by Gao et al. on three groups of patients (0 steps, 5000 steps, and 10,000 steps before the colonoscopy procedure) revealed that walking more than 10,000 steps can significantly increase BBPS compared to the other groups [23]. Another RCT study by Kim et al. on 383 patients revealed that walking exercises can significantly enhance bowel preparation, especially in non-obese patients under 65 years old and individuals without past abdominal surgery history [69]. A meta-analysis by Huang et al. found quantitative exercise before colonoscopy can increase bowel preparation in addition to reducing adverse effects of colonoscopy including vomiting, abdominal pain, and bloating [70].

In our study, younger individuals had better bowel preparation (Fig. 3). Some other studies also identified age as a risk factor for poor preparation of the bowel [53, 71, 72]. Older age is assumed to be related to higher comorbid diseases and lower colonic movements, which can lead to poorer cleansing of the colon [73–76].

In addition, in our study, smoking was another risk factor for poorer bowel preparation (Fig. 3). Other studies also confirm this finding [77, 78]. It is assumed that smokers are less likely to follow the instructions before colonoscopy precisely due to lower socioeconomic and health status [79].

Several mechanisms can justify the positive coloration between mild walking on colon cleansing before colonoscopy. One mechanism can be related to GI tract motility and secretion. GI tract movement is a complicated process resulting from interaction and coordination between multiple cell types like enteric neurons, interstitial cells of Cajal (ICC) and smooth muscle cells, which are located in the tunica vascularis layer. Throughout a process called excitation–contraction coupling, the coordinated contraction of smooth muscle cells leads to GI motility [80, 81]. It has been shown that exercise can enhance GI tract motility and glandular secretions [82–84], which leads to faster emptying of the GI tract there by a better intestinal clarity can be expected [85]. It has also been shown that intestinal clarity has a positive relationship with the time of exercising [86]. Hu et al. found that the elderly who can walk better can have better colonoscopy preparation than those who cannot walk properly [86]. Exercising can also reduce the adverse effects of solution in colonoscopy preparation, like abdominal pain and vomiting, which can be described by the effect of exercise on the enhancement of digestive blood circulation and glandular secretions. This enhancement can lead to better absorption and excretion of bowel-cleansing agents and a better tolerance of the patients [85].

Our study had some limitations. First, one expert gastroenterologist determined the BBPS. For more accurate data, we highly recommend future studies to assess BBPS by two gastroenterologists. Second, we assessed taken footsteps by a smart band which may not be accurate. We could not assess whether participants wore the band all the time before the colonoscopy. Also, we assessed the footsteps taken by individuals. We did not assess the quality of walking. We could not assess whether participants had mild exercise or moderate or severe.

## Conclusion

In conclusion, a higher number of footsteps taken especially more than 6900 can significantly enhance bowel preparation; however, walking exercise as an intervention before colonoscopy is not significantly associated with BBPS. In addition, older age and smoking could inversely affect BBPS. This finding highlights more chance of misdiagnosis of pathologic conditions in colonoscopies of elderly and smoker patients.

## Data Availability

Data from the study can be provided from Corresponding Author on reasonable request.

## Abbreviations

BBPS: Boston Bowel Preparation Scale
BMI: Body mass index
PEG: polyethene glycol sodium phosphate
OR: Odds Ratio
SD: Standard deviation
CI: Confidential interval
